# Civil society and the (im)possibilities of algorithmic contestation in migration governance

**DOI:** 10.3389/fsoc.2025.1641898

**Published:** 2025-10-08

**Authors:** Stoyanka Andreeva Eneva, Fredy Mora-Gámez

**Affiliations:** ^1^Department of Thematic Studies, Linköping University, Linköping, Sweden; ^2^Department of Science and Technology Studies, University of Vienna, Vienna, Austria

**Keywords:** data justice, contestability, contentious politics of data, algorithmic accountability, digital governance

## Abstract

The present article examines the (im)possibilities of contesting algorithmic systems in migration governance through an empirical study of civil society organizations in Sweden and Norway. It draws on qualitative methods including semi-structured interviews, document analysis and digital ethnography to examine how digital rights associations, migrant solidarity initiatives and hybrid groups focused on democratic digitalization respond to the spread of automation in welfare and migration governance. Our findings show an increasing awareness of algorithmic harms but uneven and fragmented capacities for resistance. Digital rights actors prioritize universalist frames of privacy, migrant solidarity groups focus on immediate survival needs, and hybrid organizations emphasize structural barriers to civic contestability. The discussion argues that contestability should be understood not only as a technical property of algorithmic design but also as a socio-political process that requires access to information, shared vocabularies and legitimacy for civic intervention. The study concludes that in high-trust welfare states such as Sweden and Norway, rapid digitalization combined with restrictive migration policies limits the potential for civil society contestation. The study contributes to debates on algorithmic accountability by showing how the Scandinavian experience illustrates both the potential and the limits of civil society contestation in digital governance.

## Introduction

Over the last decade, the extraction of data and the integration of automated decision-making systems and other digital technologies into migration governance have intensified, reshaping how borders are controlled, how residence and asylum applications are processed, and how people on the move are categorized and monitored. This shift is part of a broader trend toward algorithmic governance in public administration, where automated risk assessments and decision-making are increasingly applied in areas such as criminal recidivism, gender-based violence, or the allocation of social benefits (Dencik and Kaun, 2020). These systems are often introduced under the promise of greater efficiency, security, and innovation—an agenda driven by what critical scholars refer to as *techno-solutionism* and *technophilia* (Seuferling and Leurs, 2021).

However, even if these technologies are usually deployed with the aim of improving workflow in sectors marked by understaffing and administrative strain, their actual targets are often already vulnerable populations. Some examples are people in prison or beneficiaries of social welfare (Bircan and Özbilgin, 2025). People on the move, in their legal and political status as non-citizens, face an additional layer of vulnerability when targeted (Broeders and Dijstelbloem, 2018). It is in this context that migration governance has become a testing ground for some of the most aggressive and experimental technology use (Molnar, 2020; Yang et al., 2024).

Across various stages of the migration process, new technologies are used in highly contested ways. The datafication of migration governance includes border control through drones, thermal cameras, and biometric surveillance (Glouftsios and Casaglia, 2022; EuromedRights, 2024); invasive data extraction from mobile phones and digital devices (Pollozek and Passoth, 2019); automated decision-making in vital procedures such as asylum interviews, language and dialect recognition (Van der Kist, 2025), residence permit processing (Ozkul, 2023); and, increasingly, the use of predictive analytics to assess migration flows or categorize individuals by risk (Mora and Eneva, forthcoming). This type of technology use often operates in legal gray zones, enabling practices that would be considered illegal or at least unethical in other policy domains (Molnar and Gill, 2020).

Accountability is elusive, as decision-making is black-boxed and displaced from public scrutiny (Ozkul, 2023; Redden et al., 2022). But it is not only a matter of developing a transparent and accountable technology. Automated decision-making is embedded within socio-technical assemblages shaped by unequal power relations, corporate influence, and supported by public opinion trust (or a feeling of inevitability) in technological innovation. Scholars from Critical Migration Studies and Science and Technology Studies argue that historical forms of discrimination and control are intensified through technology (Valdivia and Tazzioli, 2023). In this context, technology is not merely a tool but a political actor that is reshaping the categories through which individuals are recognized as citizens or excluded. Algorithms do not simply process information; they actively participate in the construction of risk profiles and the development of policy responses. People on the move are thus reduced to data points and statistical categories, reinforcing racialized and exclusionary logics under the guise of neutrality and efficiency.

Alongside this critical scholarship, another important research line has emerged, documenting how data extraction and automated decision-making systems are contested from below. Scholars such as (Lyons et al. 2021), (Beraldo and Milan 2019), (Hintz et al. 2022), (Milan and Hintz 2013), (Treré and Bonini 2024), and (Galis et al. 2022), among others, have focused on agency, grassroots strategies of resistance, subversion, and appropriation of digital technologies by migrants, activists, and solidarity networks. Their research highlights practices such as evasion, counter-mapping, and the development of alternative infrastructures.

Building on this second line of work, we focus on the discourses and practices that critically engage with the expansion of automated decision-making systems in migration governance. We pay special attention to the ways automated decision systems are being questioned, problematized, and resisted by civil society actors. Specifically, we draw on the concept of AI contestability (Alfrink et al., 2023; Lyons et al., 2021), not just as a technical process, but as a part of a socio-technical system where claims of transparency and justice are negotiated.

We base our analysis on a study of civil society organizations in Sweden and Norway, two countries often associated with high levels of innovation, digitalization of services - including the public sector - and institutional trust (Dexe and Franke, 2020). At the same time, both countries are interesting and relevant case studies because they have introduced increasingly restrictive migration policies undermining their long-term line of international solidarity and generous asylum policies (Dahlstedt and Neergaard, 2019), combined with the use of digital technologies whose implications for migrants' rights remain underexplored. The aim of our study is to explore the position of civil society organizations in relation to the use of migration governance technologies that may reinforce existing inequalities or create new forms of vulnerability. In doing so, we analyse how different types of civil society actors in Sweden and Norway engage with the growing datafication of migration governance. We also explore what types of technical, legal, or symbolic contestability are feasible in this context.

First, the article outlines how the concepts of data justice, contestability, and the contentious politics of data inform our theoretical framework. We then outline a typology of contestation, focusing on the configurations of actors and forms of alliance-building. Finally, we analyse and discuss our case studies, which include three types of organizations: those working on digital rights, migrant rights, and democratic digitalization.

This article contributes to debates on digital governance, data justice, and migration by highlighting the conditions that shape the (im)possibility of contestability. Far from offering a representative mapping of civil society engagement in this area, our purpose is to trace the tensions, gaps, and emerging strategies that define the conditions of possibility for contestability of algorithms in migration governance.

## Data justice and contestability in migration governance

Understanding how civil society actors respond to the expansion of automated decision-making in migration governance requires attention not only to technical systems but also to the broader political and social dynamics in which these systems are embedded (Latour, 2005; Broeders and Dijstelbloem, 2018). The concept of data justice offers a powerful entry point for this analysis. Rather than focusing solely on legal safeguards or algorithmic bias, data justice draws attention to the structural conditions that shape how data is collected, interpreted, and used. It reveals how datafication is not simply a process of modernization or increased efficiency, but one that reflects and reproduces deep-rooted inequalities. As scholars such as (Sánchez-Monedero 2018) and (Sánchez-Monedero and Dencik 2022) argue, algorithmic decision-making does not affect everyone equally. It disproportionately harms those who are already marginalized and are often the subjects of intensified surveillance and categorization through opaque digital systems.

Implemented in migration governance, these systems operate mainly for migration control and restriction. Technologies used to manage mobility are rarely neutral (Tsagarousianou, 2024); however, discussions about fairness and rights in data governance often remain limited to questions of transparency, privacy, and individual accountability. This framing obscures the social justice dimensions of contesting such technologies, where the issue is not only how decisions are made, but who gets to define the terms of inclusion, access, and legitimacy.

This becomes particularly relevant when engaging with debates centering the concept of contestability (Alfrink et al., 2023; Lyons et al., 2021). Lyons et al. consider contestability to be both an end in itself, as a fundamental democracy principle and a means to an end, as an implementation tool that guarantees the democratic rights of the affected. They stress the importance of the possibility for contestation at different stages and levels, from policy-making prior to the implementation of automated decision-making systems to the design of how they function and the possibility to appeal the already-made decisions.

In a similar way, (Sarra 2020) argues that a real right to algorithmic contestation involves more than including a “human in the loop” who has limited agency and mainly validates machine-made decisions. The possibility of just appealing a decision after it has been made is also insufficient. Contestability must include the possibility of challenging the process and assumptions that led to the decision in the first place. A person affected by an automated system must be able to question how and why decisions are produced and not just whether a specific result was “correct.” Contestability, in this sense, is not a static design feature but a relational and dialogical practice. It presupposes meaningful interaction between the system and those affected by it, which is often absent in current applications of automated decision-making in migration governance.

However, technological resistance does not occur in a vacuum. In recent years, researchers have begun to map how migrants and solidarity actors respond to the datafication of borders. Drawing from social movement theory and science and technology studies, (Beraldo and Milan 2019) propose the concept of *contentious politics of data* to describe how data infrastructures are both tools of control and sites of struggle. They argue that contestation can take many forms, ranging from small acts of evasion and manipulation to collective organizing and the development of alternative infrastructures. Even individual tactics such as stepping out of digital visibility or using encryption tools can be part of broader, networked practices of resistance, particularly when shared within migrant communities or activist spaces.

## Contesting algorithmic migration governance: a typology of civil society responses

This section systematizes literature that maps diverse ways in which automated decision-making systems in migration governance are resisted, contested, or navigated by migrants and civil society actors, focusing on both the types of actors involved and the modes of action they employ. It also reflects on how existing research addresses the obstacles to contestability. In order to avoid conflating different practices, we distinguish analytically between awareness-raising, navigation, contestation, and resistance. Awareness-raising refers to activities such as public campaigns, information sharing, and digital literacy workshops that aim to broaden understanding of technological harms without necessarily challenging power holders directly. Navigation captures the coping strategies civil society actors and migrants develop to adapt to restrictive or opaque systems in order to secure rights and survival. Contestation goes a step further, involving efforts to challenge specific technologies, decisions, or policies through legal appeals, lobbying, or counter-expertise. Resistance, finally, denotes broader structural challenges to the logics underpinning algorithmic migration governance, often through coalition-building or connecting digital rights to broader struggles for social and racial justice.

While we treat these as distinct analytical categories, they often overlap in practice. Awareness-raising campaigns may also contain elements of resistance when they frame digitalization as a structural problem, while navigational strategies often evolve into contestation when coping tactics reveal systemic injustices. This fusion reflects the reality of civil society action under constrained conditions, where limited resources and urgent priorities push organizations to adopt hybrid repertoires rather than neatly separated strategies.

### From solitary navigations to collective agency

Individual strategies employed by people on the move to navigate digital surveillance are increasingly documented. These include tactics of invisibility, staying “under the radar,” and the strategic withholding or sharing of personal data. While such acts may not always appear as resistance, scholars have warned against dismissing them as passive compliance. (Tsagarousianou 2024) emphasizes the need for a nuanced understanding of agency as embedded in unequal power relations; what may appear as consent or non-resistance is often a survival strategy adopted under coercive conditions. Similarly, the concept of digital resignation (Draper and Turow, 2019) describes the feeling of helplessness that emerges when individuals face overwhelming data systems they cannot influence or escape, leading them to reluctantly submit to data extraction rather than approve it. In the context of migration governance, other urgent material risks are often prioritized over surveillance and data extraction. As (Masso et al. 2024) have shown physical violence, arbitrary detention, invasive search procedures are logically perceived as more immediate threats to bodily autonomy and even life.

Research shows that invisibility itself can be a strategic, and often collective, practice. Migrants may deliberately minimize their digital footprint as a collective knowledge tactic to reduce exposure to surveillance. (Amelung and Galis 2023) describe how, especially during phases of legal or existential precarity, migrants adopt “under the radar” tactics not simply to remain hidden, but to maximize their chances of continuing their migration project. These strategies are not merely defensive or isolated; they are embedded in informal support structures and collective routines of navigation. (Talvitie-Lamberg et al. 2024) similarly argue that invisibility and withdrawal from certain online spaces such as social media platforms can be a form of spatial self-determination and a means of claiming autonomy. Even when these practices do not generate formal resistance, they reshape how space, data, and visibility are negotiated by those navigating hostile digital terrains.

Together with (and sometimes in contradiction with) avoiding visibility, building forms of care, support, and knowledge exchange is another strategy that enables migrants to navigate hostile systems. Through various online tools (often in a limited or anonymized way) migrants build networks of digital care (Palmberger, 2022), where they share valuable knowledge on border-crossing. In many cases, these networks emerge from the need to stay mobile, informed, and safe. This includes the use of mainstream platforms and social networks in strategic ways such as private groups, encrypted chats, or coded language. The creation of this type of safe digital spaces may be temporary, fragile, or invisible to outsiders. Knowledge and support circulate horizontally in this networks, often outside traditional hierarchies of leadership or institutional legitimacy (Ponzanesi and Leurs, 2022). These networks can also serve as bridges to different civil society actors who could get involved.

In this sense, coalition-building is a vital form of collective agency. Although often precarious and uneven, coalitions between migrant-led and/or migrant solidarity groups, digital rights organizations, legal advocacy actors, and academic allies have become essential for amplifying contestation efforts. These alliances bridge gaps in expertise and access, connecting those with lived experience of digital border control to those with legal, technical, or institutional knowledge (Galis et al., 2022). Coalitions enable coordinated action across different fields of struggle, enhance legitimacy, and create opportunities to engage policymakers and public discourse from a more unified front. While fragile and shaped by unequal resources, coalition-building offers a promising path toward sustained and multisectoral forms of contestability (Galis and Neumayer, 2016; Galis and Summerton, 2017).

Coalitions between civil society actors have also developed at the international level, working through advocacy, lobbying, and awareness-raising. Some of the most relevant examples include European Digital Rights (EDRI*)*, which focuses predominantly on digital rights, while also being attentive to the ways in which different social groups are exposed to varying levels of risk of violating them. Another relevant coalition is the Platform for International Cooperation on Undocumented Migrants (PICUM), mainly focusing on migrants' rights but also following closely the way migrants are policed and categorized by new digital technologies. Lastly, *Protect, not Surveil* is a coalition that bridges intersectional analysis of vulnerability with a focus on technology and expertise. The coalition has been specifically created to dispute legal and policy changes that will affect migrants and digital rights at the EU level, such as the EU AI Act.

### Evasion, structured campaigns, and strategic action: a continuum

The second analytical axis concerns the forms of action these actors undertake, which range from evasive and everyday tactics to structured campaigns and legal interventions. Rather than treating resistance as a binary of acceptance or confrontation, recent literature invites us to view it as a continuum of practices situated within complex social and technological conditions.

This continuum is helpfully illustrated by the concept of ResTech—resistance technologies—developed by (Jiva et al. 2025). Their typology categorizes digital and technological responses according to their intent, design, and function, showing how diverse forms of engagement co-exist and interact. ResTech initiatives include: responsible technologies, which emphasize ethical design practices and data protection; responsive technologies, which address urgent community needs through practical tools; resilient technologies, which support community-building and long-term self-advocacy and, lastly, resistance technologies, which confront exclusionary systems and directly challenge digital governance through visibility, empowerment, and political claims-making.

This framework supports the argument that not all technologies used by or for migrants are explicitly designed as resistance tools. Some are low-tech, locally developed, and focused on immediate needs, while others are more strategic, aiming to shift power relations or influence discourse. Their shared focus on community orientation, harm reduction, and situated knowledge offers an alternative to top-down technological solutions, underscoring the multiplicity of contestation practices.

An example of this complexity is the concept of digital migrant spaces (Makrygianni et al., 2022) which refers to online and offline environments where migrants and their allies engage in various forms of communication. These spaces enable migrants to access knowledge, support each other, and counter mainstream narratives that criminalize mobility. Rather than functioning as traditional activist platforms, the digital migrant spaces create hybrid forms of solidarity that blend care, tactical evasion, and collective learning.

Similarly, the practice of counter-information (Makrygianni and Galis, 2022) highlights the importance of challenging dominant narratives and misrepresentations of migration by creating alternative information flows. This includes debunking misleading official claims (e.g., denial of pushbacks) and producing knowledge from the perspective of migrant experiences. Counter-information is not just about speaking back; it is also about reclaiming space and narrative power in the face of hostile bordering practices and racialized media discourses.

Complementing these perspectives and engaging with the specific ways algorithms affect the discourses and practices of social movements, (Treré and Bonini 2024) propose a typology of algorithmic resistance, detailing how activists interact with algorithmic systems in different ways. The most relevant tactics they have identified are evasion (avoiding surveillance or bypassing algorithmic detection), appropriation (creatively reusing digital tools for their own purposes) and hijacking (manipulating algorithms to subvert their original function). This classification provides analytical clarity to the numerous informal and creative tactics developed across different movements. It highlights that resistance can occur both through silence and visibility, through absence and disruption.

Beyond grassroots and informal strategies, another crucial layer of contestation involves institutional and legal engagement, particularly through strategic litigation, policy influence, and the production of actionable knowledge. These forms of contestation are often pursued by civil society organizations that have established positions within national or European advocacy ecosystems and engage directly with legal or political institutions.

(Palmiotto and Ozkul 2024) conceptualize strategic litigation as part of a multi-step process similar to climbing a wall: slow, incremental, and layered with obstacles. Drawing on case studies of legal action against opaque algorithmic systems, their work emphasizes the foundational role of transparency claims, legal expertise, and alliance-building. The process often begins with gaining or seeking access to information, followed by the development of technical and legal arguments, and is sustained by the co-production of knowledge among affected individuals, legal advocates, and civil society organizations. Central to this process is the creation of coalitions, often fragile but essential for developing legitimacy and leveraging the broader public discourse. While successful litigation is rare and resource-intensive, it can generate important precedents. Moreover, this type of intervention is only part of a broader ecology of contestation, and its success often depends on the groundwork laid by awareness-raising and coalition-building activities.

Indeed, awareness-raising has emerged as one of the most widespread and adaptable forms of civil society contestation, particularly in Europe. This includes organizing public events, publishing reports, facilitating knowledge exchange workshops, and engaging with media and academic platforms. These activities are crucial in generating counter-discourses and positioning organizations as legitimate stakeholders in debates around migration and technology. Their credibility often stems from dual roles: as service providers, community actors, and public communicators. In some cases, individuals within these organizations also work as researchers, journalists, or legal experts, giving them rare access to both institutional and grassroots spaces and the opportunity to produce a valuable situated knowledge.

Several organizations exemplify this multi-positioned engagement. In Spain, *AlgoRace* combines public campaigning with coalition-building and produces in-depth reports on racializing algorithmic governance, while *LaFede* focuses on local AI initiatives, movement-building between human rights and digital rights organizations and making efforts to introduce a critical discourse into a media narrative dominated by the tech industry. In the Netherlands, *Bits of Freedom* and the *Racism and Technology Center* link digital rights with anti-racist advocacy, engaging in both awareness-raising and policy lobbying. In Greece, *Homo Digitalis* operates at the intersection of legal action and public communication, combining strategic litigation with education and campaigning. Meanwhile, the *Migrants' Rights Network* in the UK develops community-based workshops focused on horizontal knowledge exchange between activists and migrants addressing the use of technology in migration governance and its direct impact on people's lives. Their work highlights a horizontal knowledge production model that values the lived expertise of migrants and treats them not just as beneficiaries but as active participants.

These examples show that awareness-raising is rarely a standalone activity. It often functions as a platform for coalition-building between frequently disconnected actors, especially in national contexts where migrant rights and digital rights organizations operate in separate spheres. While technical expertise often resides in digital rights NGOs, and a different set of actors carries out migrant solidarity work, awareness-raising initiatives can act as connective tissue and foster mutual learning and laying the groundwork for joint action.

In this sense, awareness-raising is both a practice of resistance and a precondition for more confrontational strategies. It plays a key role in shaping public narratives, legitimizing new actor constellations, and making visible both the harms of algorithmic migration governance and the capacity for civil society to contest them, even when legal, technical, and political barriers remain high.

## Case selection: Sweden and Norway

Sweden and Norway offer a particularly relevant lens for studying civil society responses to algorithmic migration governance. Both countries are global leaders in the digitalization of welfare and administrative systems, and their national strategies actively promote not only digitalization but also the use of AI and automated decision-making across sectors. These developments are framed through a strong techno-optimist lens, where innovation is seen as inherently positive, and are supported by well-developed public agencies, academic institutions, and private actors. At the same time, ethical oversight bodies and regulatory frameworks exist, yet civil society actors with critical perspectives remain relatively marginal within this landscape.

Migration policy in both countries has undergone a marked restrictive turn since 2015. This shift led to tightened asylum rules, fewer opportunities for regularization, and reduced possibilities for migrants to build long-term life projects in the host country (Hagelund, 2020; Hellström, 2021). These restrictive trajectories are increasingly mediated through technologies such as predictive algorithms, automated resettlement systems, and data extraction from mobile devices (Ozkul, 2023). Although Sweden and Norway differ in their welfare regimes and reception histories, both are high-trust societies where civil society has historically operated in close dialogue with the state (Trägårdh, 2022; Enjolras and Strømsnes, 2018; Peterson et al., 2018). In migration-related issues, however, negotiation and cooperation with authorities tend to predominate over confrontation (Micinski and Jones, 2022). This combination—rapid digitalization, restrictive migration policies, and a civic culture grounded in consensus—creates a unique configuration that shapes how contestation becomes possible.

At the same time, important divergences make them suitable for comparative analysis. Sweden historically pursued a more generous reception and integration model, which fostered the development of stronger migrant solidarity networks—although these have been increasingly defunded in recent years. Norway, by contrast, has long maintained a more restrictive asylum and family reunification policy (Brochmann and Hagelund, 2012), and civil society advocacy is more often channeled through professionalized NGOs and broader human rights frameworks. Digitalization strategies also differ: Sweden has explicitly embraced digital innovation as part of asylum and welfare reform, whereas Norway combines digitalization with a comparatively stronger emphasis on data protection and regulatory oversight.

Even though we were interested in a Nordic comparative perspective, we decided not to focus on Denmark and Finland for various reasons. Denmark constitutes a regional outlier due to its exceptionally restrictive migration regime and a more polarized civil society landscape. At the same time, Finland, although engaged in digitalization, plays a less central role in shaping European debates on migration governance. Sweden and Norway thus provide a balanced comparison: similar enough to generate meaningful cross-case insights, yet distinct enough to reveal how different welfare and migration regimes condition civil society responses to algorithmic systems.

In what follows, we briefly explore how the above-described conditions shape the way civil society organizations engage (or struggle to engage) with migration surveillance, data extraction and the growing use of automated systems in migration governance.

## Methodology

We adopt a qualitative, multi-sited research design to investigate how civil society organizations in Sweden and Norway engage with the increasing digitalization of migration governance. This approach allows us to capture both the transnational dynamics of digital rights advocacy and the situated ways in which organizations in specific national contexts frame and respond to digital technologies in migration control. By combining digital ethnography, semi-structured interviews, and document analysis, the study aims to illuminate the intersection of digital rights and migrant rights struggles across various sites of civil society activity.

## Interviews and data analysis

Our interview sample was constructed through a purposive selection process. We began by mapping organizations in Sweden and Norway engaged with digital rights advocacy and migrant solidarity, with specific attention to groups at the intersection of these two domains. Beyond organizational type, we also considered the kinds of activities and events they organized or participated in, especially those linking debates on migration with broader questions of digital governance.

In total, we conducted four semi-structured interviews between October and December 2024. Three interviewees were men, and one was a woman. One participant was employed in a professional capacity within their organization, while the remaining three were active as volunteers. The interviews were conducted remotely, recorded with consent, transcribed verbatim, and subsequently anonymized to ensure confidentiality. The participants provided written informed consent to participate in this study. The interviews were designed to explore how organizations perceive and respond to the use of digital technologies in migration governance, what obstacles they encounter in contesting these systems, and how they position themselves in broader civil society landscapes. The sample includes three broad types of organizations: digital rights organizations, migrant rights and antiracist organizations, and organizations focused on economic inequalities and democratic digitalization. The organizations included in this study, whether observed online or represented by interview participants, are listed in Appendix.

We conducted thematic analysis using the qualitative data analysis software CATMA, combining thematic coding with an abductive approach. This allowed us to iteratively connect emerging themes from the interviews with our theoretical framework of data justice, contestability, and contentious politics.

## Digital observation and ethnography

We also focused on the online presence and activities of civil society organizations in Sweden and Norway. The selection process began with the observation of large-scale international campaigns and coalitions at the intersection of digital rights and migrant rights, such as *Protect Not Surveil* and the European Digital Rights (EDRi) network. By tracing participation in these campaigns through open letters, press releases, and coalition websites, we identified Scandinavian organizations that were either signatories or partners.

During this process, it became apparent that Scandinavian organizations were underrepresented in international coalitions compared to their European counterparts. This finding itself became an empirical entry point, as it highlighted the specificity of the Scandinavian landscape of digital and migrant rights advocacy. This is why we started our observation by those organizations that were present in such transnational initiatives and campaigns.

The digital observation included a systematic review of organizational websites, newsletters, and social media channels (Twitter/X, Facebook, Instagram), as well as attendance at online events and webinars. The aim was not to analyze individual users or private data but to capture organizational narratives, strategies, and modes of coalition-building in the Scandinavian context. While this material was publicly available and did not involve interaction with individual users, ethical considerations for digital research were taken seriously (Markham and Buchanan, 2015): no personal data or user profiles were collected or analyzed, and the focus remained on organizational narratives, communication strategies, and engagement patterns.

Digital ethnography complemented the interviews by providing insights into how organizations publicly frame their engagement with digitalization, discrimination, and migration. Since these are publicly available materials, organizations may be identifiable; however, in order to maintain consistency with the anonymization strategy applied to interview data, we refer to them in analytical rather than descriptive terms. This triangulation allowed us to capture not only internal perceptions but also outward-facing strategies and silences, thereby contextualizing the interview data within broader communicative practices.

In parallel, we examined reports and policy briefs published by civil society actors such as Amnesty International, AlgoRace, Bits of Freedom, PICUM, Protect not Surveil, Algorithm Watch and the Racism and Technology Center. These sources were selected because of their influence in European debates on AI and migration, and they served as important secondary data sources in order to situate the Scandinavian material within broader transnational discourses on digital rights and data justice.

## Digital rights, reproduction of vulnerabilities, and social justice: civil society responses to datafication

The empirical material presented in this section draws on our analysis of three types of organizations engaged with the datafication trends and migrants' rights in Sweden and Norway. The people we interviewed and the organizations whose online presence we observed work mainly through awareness-raising, policy advocacy, and knowledge dissemination. While some members of these organizations are migrants themselves, they do not currently experience digital vulnerability in the same way as the people they aim to support.

Referring to the typologies discussed earlier in the literature review and relying on the available empirical material, our focus is less on tactics of invisibility or direct resistance by affected individuals, but rather on how civil society actors raise awareness about surveillance, analyze policy developments, and attempt to influence them. Their efforts are directed to strengthening civil society participation and building alliances across organizations that focus on intersecting inequalities. However, limited time, resources, and organizational capacity often prevent this from fully materializing.

We identified three types of civil society organizations closely related to the topics of digital and/or migrant rights, whose priorities can be categorized as follows. The first one is related to a strong defense of privacy and individual data ownership, along with raising awareness about digital rights as a form of human rights. The second type of organization focuses on the growing vulnerability of migrants and the alignment of increasingly restrictive mainstream migration policies with far-right agendas. In this context, digital concerns often remain in the background of their focus, although there is a clear consensus that they should not be ignored. Lastly, we identified a broader political perspective, concerned with the digitalization of public services as part of a wider process of privatization, shrinking civic space, and the consolidation of corporate power. Here, digitalization is framed in connection to long-term commitments to social justice.

## Digital rights, for everyone?

In order to focus on the defense and promotion of digital rights and differentiated access to them, we examined at organizations with a long-standing tradition of promoting and defending individual privacy and data ownership, often recognized for their involvement in open-source practices and defense of Creative Commons licenses. Their profile is also related to periodic mobilizations for online privacy, digital sovereignty, and the threats posed by surveillance systems. These organizations are often connected to broader international networks that link digital rights and migration, with a strong focus on surveillance infrastructures and their implications for marginalized communities, such as EDRI.

Their activities are primarily focused on awareness-raising, advocating for policy change and providing individuals with tools and advice to protect their privacy. Through different communication channels, including website content and social media presence they advocate for individual rights from the perspective of universal needs. The campaigns in which they participate often target tech-savvy users or those open to digital literacy initiatives, confirming the observations of (Dencik and Sanchez-Monedero 2022) regarding the disconnection between social justice and technical labor in activism. Through the conducted interviews, Björn, a member of the board of organization O1, shared that they also actively contribute to the Tor network, helping users achieve privacy through decentralized and anonymous participation.

Participation in organizational networks and international alliances, such as EDRi provides access to updated knowledge on digital rights and related social issues (for instance, vulnerable groups' rights, including migrants) even when these topics are not their central focus. Björn explained that EDRi's annual meeting is an important source of information for them and that they typically send a delegate to attend. Similarly, in the case of O_2_, analyzed through their website and social media channels, we found regular reposting of materials from EDRI, highlighting the value of these networks for their work.

O1's awareness-raising campaigns are mainly directed against the surveillance of private communications and, more recently, advocating for the use of open-source, publicly developed and transparent software in public administration has also been added to their priorities. Their long-term work on specific initiatives follows a step-by-step logic: identifying a specific issue, gathering information, conducting research, contacting journalists and politicians, and developing functional prototypes as alternative solutions. Their strategy combines technical expertise with efforts to mobilize public attention and influence actors with decision-making power, operating across micro and meso levels.

### Digital rights as a matter of democracy and human rights

The interviewees stated that their primary concern is digital rights, focusing especially on privacy and data ownership, with the aim of producing critical discourse and preventing or reversing surveillance practices.

They express a firm conviction that privacy, control over one's own data, and freedom from coercive digital identification systems are human rights that are increasingly important in a datafied world. They also show awareness that these rights are not equally distributed and their violation does not affect different social groups in the same way:

*We are trying to achieve something that is good for everyone. General things like privacy should be seen as a human right, and it should be something that everyone can have. It should not be strange. It should not be restricted to certain groups. But having said that, it's also true that there are certain groups of people for whom it becomes more important, even critical (Björn, O1)*.

O2′s online material also reflects this tension. While its website and campaigns (e.g., Reclaim Your Face) highlight how surveillance threatens all citizens, its coverage of migration-related surveillance is minimal. References to changes in asylum law or data protection in migration policy are posted as neutral news items, without comments or critical reflection. This silence contrasts with their vocal stance on general digital rights issues, suggesting that migrant-specific risks remain underarticulated in their public framing.

Zooming out, Björn presents a broader picture that encompasses not only individual control over personal data but also more significant concerns regarding democratic accountability. In his view, the problem is exacerbated by the increasing reliance of public administrations on private digital infrastructures. It is not just individuals who are coerced into giving their data to major search engines, social media platforms, and cloud storage providers. Governments, too, are outsourcing their technological infrastructure to the same actors. Instead of developing public, transparent systems, state agencies often subsidize and purchase services from private corporations, thus losing control over critical systems.

*They lose the possibility to have control over what they are doing. And it's terrible from a democracy perspective… if we compare this to an alternative reality where this government agency would create their own system and they could show people, the citizens: “Okay, this is our system, this is how the system works. Please give us feedback on how we do this”. This is a democracy (Björn, O1)*.

This concern is echoed on the O_2_ website, where public campaigns focus on achieving digital sovereignty calling for greater transparency, independence, and public ownership in the development and implementation of digital systems used by state institutions.

At the same time, Björn pointed to a contradiction: although there is greater public awareness of data extraction and surveillance, this has not led to increased resistance. Instead, there is a generalized perception of digital resignation (Draper and Turow, 2019). Furthermore, despite the privatization of data infrastructure, the increasing surveillance by both states and private platforms, and the shrinking role of the public sector, social trust in institutions remains remarkably stable.

### Everyone is surveilled but some are more surveilled than others

Despite the strong emphasis on surveillance as something that affects virtually everyone, the interviewee showed awareness that it does not affect everyone equally and its impact is stronger for already vulnerable and/or marginalized groups. As Björn explained, many people (especially those perceived as “regular citizens”) are aware of being surveilled but may not feel directly harmed by it, since they are not seen as suspicious or threatening by authorities. In contrast, individuals who are racialized or otherwise marked as “other” are far more likely to be targeted by surveillance systems in ways that carry real consequences. The perceived indifference of the majority toward surveillance, he suggests, is tied to citizenship privilege and the normalization of selective scrutiny.

Overall, the interview with Björn offered a rich set of reflections on the relationship between anti-migration sentiment and increasingly restrictive policies, as well as the use of surveillance technologies that represent a danger for human rights. In Björn's opinion, the authoritative turn in governance is closely tied to the way migration is managed:

*There are a lot of legal changes in the direction of more surveillance and more forcing people to do more things and reducing the requirements for certain police actions. And there is also a strong, let's say, anti-immigrant attitude that it's not prioritizing human rights. Human rights are removed and we are getting a more authoritarian system instead. (…) And I think that the reason it's moving in that direction, it's not explicitly said, but there's a lot of indications that it has to do with migration (Björn, O1)*.

Both the interview and the digital observation show that digital rights organizations' increasing awareness of the intersection between digital rights and human rights restrictions. For example, 06 a Swedish collective grounded in socialist, feminist, and anti-racist principles whose website we observed, frames its work explicitly in terms of “self-defense in the age of digital fascism.” Through public workshops, coalition-building and joint declarations, they illustrate that digital rights can also be mobilized as part of a broader emancipatory project. Digital ethnography of their online presence reveals that they act as connectors, offering training, allyship, and advocacy that bridges technical concerns with struggles against racism and social exclusion.

In conclusion, this first type of organizations could be considered key actors within a broad ResTech ecosystem, offering infrastructure, knowledge, and a critical perspective. They demonstrate awareness of how surveillance technologies impact different communities in different ways, yet their public discourse and activities remain directed toward digital and human rights in a universal manner (“it is something that affects everybody”). Their primary audiences are tech-savvy individuals seeking privacy (e.g., through Tor), the general public (via campaigns like *Reclaim Your Face*), and public authorities, whom they address in order to promote responsible technology use. Despite a clear understanding of discrimination and its consequences, practical engagement with migrant communities is limited, and no direct collaboration initiatives were mentioned. Interestingly, despite the interviewee's technical expertise, his insights into how migrants are affected by discrimination were mostly framed in terms of analog human rights violations and pre-existing societal biases rather than specific consequences of datafication.

## Migrant solidarity organizations: between analog urgencies and digital awareness

The second profile we turned our attention to is migrants' rights and antiracist advocacy associations. While violation of migrants' rights through digital technologies is often not their main focus, they are aware of the emerging risks related to datafication, surveillance, and algorithmic governance—particularly as these affect disproportionately racialized and migrant populations. The information about this type of cases often comes from their everyday practice of supporting individuals facing discrimination, as well as from information shared through international civil society networks, such as PICUM. However, their capacity to engage directly with these issues is limited, both by resource constraints and by the overwhelming urgency of addressing more immediate, “analog” injustices. Reports on digital discrimination are primarily produced by larger international organizations with dedicated research capacities, such as Amnesty International (2024).

O3 is a well-established Norwegian antiracist organization with over 40 years of history. It has a clear internal structure, with departments dedicated to various areas such as youth, employment, discrimination, and far-right extremism. The organization plays a leadership role in national antiracist networks and participates in supranational organizations like PICUM. Community-building is central to their work, organizing seminars, trainings, and regular coalition-building meetings with other civil society actors. Their activities also target the general public through campaigns, awareness-raising events, and public advocacy.

Digital observation was helpful for building this profile. On social media, O_3_ is highly visible in campaigns against hate speech, Islamophobia, and unequal refugee treatment. While migration and racism are central topics, technology-related issues rarely appear as standalone themes. Instead, O3′s engagement with algorithmic discrimination primarily surfaces in collaborative events with partners, such as O4. For example, in a recent public debate on AI and discrimination, O3 contributed with a racial justice perspective, alongside digital rights and equality institutions. This suggests that their entry points into digitalization debates occur mainly through coalition-building rather than internal agenda-setting.

### Restrictive migration policies and reduced mobilization capacity

One of the main concerns expressed in the interview with Anna, a senior staff member of 03, is the increasingly restrictive direction of migration policy in Norway and the normalization of xenophobic rhetoric, which has spread beyond the far-right. She describes the current activist landscape through the concept of *activist fatigue*. During the summer of 2015, the arrival of refugees caused widespread solidarity and enthusiasm to help. Many new activists entered the field, but after a few months a growing sense of defeat took hold. As it became clear that most refugees would not be able to regularize their status and migration policies hardened, the movement's energy declined. The pandemic further disrupted mobilization efforts. At the same time, stricter asylum policies led to fewer people arriving and settling in Norway, which did not resolve the broader challenges of forced migration but instead redirected migratory routes and made these crises more invisible in the Norwegian context. This shift made it even harder to draw media and public attention to solidarity efforts.

While the organization works broadly on discrimination, including in employment, youth, and hate speech, the digital dimension of discrimination is not currently central to its work. However, they expressed awareness that these issues are gaining importance and acknowledged the need to engage more systematically, ideally in collaboration with other actors.

### Technology: present but not central

Through coalition-building and joint work with institutional actors, the organization has become aware of certain uses of technology and automated decision-making that may negatively affect migrants and minority groups. Their primary concerns are related to unequal treatment by public administration, issues often perceived as bureaucratic or institutional rather than technological.

*We're more worried about how they delegate power to people down in the system. For example, it's no longer a lawyer or someone with a legal background treating the cases. They delegate it to someone working below them. That's on the on the appeal side of it, if you get a negative decision and you appeal (Anna, O3)*.

Their strong links to trade unions and their department's work on employment issues also give them insight into how technology may reinforce discrimination in the labor market.

*I know that they use technological solutions, like video interviews instead of face-to-face ones. Our department working on aspects of work life is worried about interview processes or application processes where AI is used. If you answer so and so in the interview, some kind of AI could be used to evaluate it. And they worry that this will affect people with an immigrant background more than others (Anna, O3)*.

On the other hand, through participation in projects involving state actors, for instance, the police, they have gained insight into the transposition of EU legislation at the national level, including rules governing data extraction, mobile phone searches, and the storage of migrants' data in databases like Eurodac.

07, a Swedish network for asylum groups offers another example of how migrant solidarity organizations navigate asylum seekers different needs and problems. With a strong digital presence, its website and Facebook accounts show that it serves as a hub of expertise for asylum seekers, volunteers, and lawyers, providing guides, legal information, and updates on migration policies. Much like O3 in Norway, its resources primarily target *analog urgencies* as appeals, deportations, and everyday struggles in the asylum system rather than issues of digital surveillance or algorithmic discrimination. In this sense, 07 exemplifies how migrants' rights organizations act as knowledge brokers while remaining relatively disconnected from digital rights debates. A similar pattern is visible in grassroots movements such as 08 in Stockholm and Malmö, whose social media activity revolves around mobilizing housing, legal aid, and community support. These groups build solidarity through direct action and community-building and the absence of explicit attention to digital rights does not reflect unawareness, but rather the overwhelming immediacy of other struggles. Yet, the very reliance on digital platforms for coordination and visibility highlights the paradox. While digital infrastructures are indispensable to their activism, their implications for migrant surveillance and discrimination remain at the margins of organizational agendas.

### Coalition-building to address multi-crises

O3 actively participates in national and supranational civil society networks. European-level coalitions, particularly those with more resources dedicated to research, dissemination, and monitoring legislative developments, are crucial for staying informed.

*We don't have the capacity nor the knowledge to follow the development on the EU level. But PICUM has been following the negotiations on the Migration Pact very closely. So, they've been keeping us posted on the recent developments for a couple of years already. The criticism of the pact is massive amongst the European organizations. I very clearly see how it can make things worse especially on the external border. When it comes to the screening and the detaining of everyone crossing the external European border, but also the part of the pact referring to the internal screening obligation, because we do work a lot on ethnic profiling (Anna, O3)*.

National networks of activism can also be reactivated and expanded. For Anna, coalition-building is not just about joint action, but also about setting shared priorities and developing collective knowledge. She expressed that a joint event with O4, organized by the latter, was useful for her and her organization not only because of gaining knowledge about recent technological developments related to discrimination, but also because it allowed her to realize that she could contribute with a different perspective.

*O4 called and asked us if we wanted to participate in an event with them. I initially said, well, we don't work on this. And then I realized, well, we don't work on it directly, but we know it's a problem and we know it's more problematic when you look at it from a discrimination perspective. And this is because of the experience I have from other European organizations or the development on the EU level (…) I also realized during the event that we need to understand the concept better. And we need to follow more closely on the development also in Norway (Anna, O3)*.

Much like in the work of (Palmiotto and Ozkul 2024), this case shows how capacity-building and coalition-building often go hand in hand:

“*Now I know that there are people in O4 who have this expertise and we should cooperate more closely. Also, the Ombudsman has some projects working on it that we should follow the more closely” (Anna, O3)*.

In summary, the organization navigates a position of ambivalence. Its members are aware of the potential for technology to deepen migrant vulnerability, limit access to services, erode privacy, and expand surveillance, but they are also facing limitations, mostly related to technical expertise, human and economic resource availability:

“*I think we're kind of caught off guard. I have the impression that many of our type of organizations doesn't really work on this and haven't really had the resources or the capacity to, to understand the scope of it” (Anna, O3)*.

Migrant solidarity and antiracist organizations are thus relatively informed about algorithmic discrimination but tend to position themselves more on the receiving end of information and knowledge than as expert voices. They are aware of the risks of surveillance and also of not yet being equipped to act on it. The lack of technical expertise often contributes to a sense of disempowerment, leading to the de-prioritization of digital issues:

“*It makes it more difficult that the organizations that do work on it are not the people we usually cooperate with. So, we have to get the knowledge. We also have to find new people to cooperate with and it's competing with other issues*” *(Anna, O3)*.

But it is not only about expertise. Lack of resources is one of the most serious problems of migrant solidarity and antiracist organizations. Due to the prolonged activist fatigue, together with the increasing criminalization of migration and the increasingly restrictive policies, the lack of volunteers and activists and the exhaustion of the remaining ones is palpable:

*There are many acute situations already that we have to address. Then we would have to see where do we spend our resources, because I don't see more opportunities for funding in the near future. The tendency is that getting funding is getting more difficult, even for the activities we have already going now (Anna, O3)*.

As a result, the migrant solidarity organizations operate primarily within the realm of awareness-raising and sometimes service provision, but from a relatively low-profile position. They remain alert to the growing importance of technology in migration governance and the increasing danger of human rights' violation and aware of the need to prepare in order to avoid being caught off guard again.

This reliance on external expertise is also evident in their online communication: technology-related content typically reproduces institutional reports rather than original statements. This underlines their position “on the receiving end of knowledge,” where digital risks are acknowledged but not systematically addressed.

## Democratic digitalization in practice: bridging advocacy, expertise, and structural critique

The third case study focuses on a hybrid organization that is both established and evolving, currently reshaping its structure and agenda. O4 is the Norwegian branch of a transnational organization with a long history of mobilization around global inequalities, particularly those linked to economic justice, such as tax havens. In recent years, the organization has broadened its focus, and one of its most active working groups today is dedicated to democratic digitalization. While it has a broad membership base, interviewees noted that there is limited engagement in events and regular activities.

The organization operates through a well-developed internal system of research, lobbying, and political communication. One of its strongest claims relates to the structural conditions that make technological contestability (im)possible. The organization strongly criticizes the lack of public funding and accessibility of civil society actors to decision-making spaces. We interviewed Christian (Democratic digitalization group coordinator) and Mario (CEO), who shared their understanding of the concept of contestability not simply as a question of technical issue or legal access to appeal mechanisms, but as a broader question of democracy: who can participate in shaping technology, and in what circumstances?

As (Palmiotto and Ozkul 2024) suggest, expertise-building is a key step in “climing the wall” of technology contestation. O4's democratic digitalization working group is an example: composed of technically skilled individuals who are tightly coordinated with the organization's board. This internal structure enables a flow of technical knowledge into organizational strategy and external advocacy. As Mario said, what is at stake is not only the erosion of privacy in cases of surveillance but also a deeper reconfiguration of how economic value is generated:

*There is a total transformation about how value is generated. During the next stage of society we are entering in, value will be generated mainly through data and control over data. It will be the main economic resource. We could call this the datafication of the economy (Mario, O4)*.

This emphasis is consistent with their digital presence. An active Facebook page and organizational blog frequently discuss democratic digitalization in relation to global inequalities, neoliberalism, and climate justice. While engagement is low, the content positions O4 as a bridge-builder, translating technical debates into broader social struggles. In recent years, their CEO has published op-eds and participated in parliamentary hearings, confirming their dual strategy of grassroots awareness-raising and institutional advocacy.

### Organizational strategy and movement-building

O4 combines an efficient internal structure with a strategic approach for alliance-building and public advocacy. Internally, coordination between technical groups and political strategists is key. As Mario explained, they regularly analyze proposed legislation through technical readings in the different working groups, which are then translated into political recommendations and advocacy messages. In parallel, they run public awareness campaigns and facilitate collective learning processes.

Their role in horizontal coalition-building is also significant. The organization positions itself as a mediator and knowledge-sharing actor in national and international networks. They aim to empower other civil society actors who are often less equipped to respond to rapidly evolving digital developments, in order to build collective responses. As Mario explained, the digital realm is perceived as a new and highly complex field, where even private actors and state institutions struggle to stay informed and updated. For civil society, the barriers are even higher. In this sense, O4 sees its mission not only as producing expertise, but also as helping form a collective civil society front capable of challenging the power and lobbying capacity of digital corporations.

At the same time, they point to a lack of institutional mechanisms and funding schemes for civil society participation. In their view, this exclusion is particularly evident in the structure and priorities of the newly established Ministry of Digitalization in Norway, which has failed to include civil society actors in its policymaking process meaningfully. This, they argue, makes the digitalization agenda one of the most exclusionary policy areas in current Norwegian politics, where civil society usually plays an important role.

### The politics of technology: what is the real problem?

While the organization is aware of risks associated with discrimination, technical opacity, and black-boxed algorithms, its critique of digitalization extends beyond these points. Their central concern lies in how digital infrastructures are embedded in broader neoliberal logics, and how they serve as vehicles for privatization, inequality, and depoliticization. They advocate for a vision of democratic digitalization that resists market-driven imperatives and instead frames digital transformation as a matter of public policy. As Mario noted, digitalization should not be treated as an inevitable or inherently positive development, but as a political domain that must be governed through public accountability and social justice principles.

For them, making claims that connect digitalization to other policy fields such as environmental protection, labor rights, and anti-discrimination is a deliberate strategy to foster public engagement and politicize these topics.

This perspective has important implications for contestability. As discussed earlier in the article, high levels of institutional trust may limit the mobilization capacity of social movements to question automated systems. In this case, another barrier is added: techno-optimism. Christian describes it as a pervasive attitude across all levels of public policy, where digitalization is assumed to be beneficial by default. However, he also admits that there is a shared understanding of concern of technological bias in expert and policy-making circles, even if he argues that the way the problem is defined is not accurate and consequently, neither are the possible solutions:

*Norway has thrown a lot of research money on so-called responsible AI and explainable AI. In that way, they are open about the fact that they know that AI, its current state can't really be used in a responsible way. So, I feel like that implies in a way that at least some politicians are aware of these things. I mean, I still kind of reject the solution that you take a bad technology and just fix it by making it responsible afterwards (Christian, O4)*.

Although the organization does not work specifically on migration or asylum systems, they are attentive to how digitalization intersects with these areas in terms of public policy. They express concern about the general risks posed by surveillance technologies and about the cognitive and infrastructural barriers faced by migrants—especially in a context where anti-immigration discourse is intensifying. As Mario stated “*The vulnerability of migrants is much higher, we have to admit that. And the context of increasing anti-migration discourses makes it much more dangerous” (Mario, O4)*.

## Discussion: the (im)possibilities of contestability

This article examined how civil society organizations in Sweden and Norway engage with the expanding datafication of public services and, particularly in the context of migration governance. It analyzed three different organizational types: digital rights associations, migrant solidarity groups, and miscellaneous topics organizations currently focused on democratic digitalization.

A central finding is that the capacity for meaningful contestation remains limited, unevenly distributed, and fragmented, despite an increasing awareness of surveillance, data extraction, and automated decision-making systems, as well as the different ways these affect various social groups. Whereas data practices that put at risk migrants' (digital) rights and increment vulnerability are getting more and more common, the defense of digital rights and migrant rights as part of the activist organizations landscape does not grow in the same exponential way. This is not due to a lack of concern, but rather to a structural misalignment of resources, expertise, and organizational focus, and it is connected to the specific development of civil society political culture in the region.

Digital rights organizations, while vocal and technically competent, operate within a paradigm of universalist discourses (surveillance as something that affects everybody) that privileges individual privacy and tech-savvy users. Their campaigns often frame data protection as a human right, but the imagined subject is typically an empowered individual who is familiar with technology and can easily participate in digital self-defense. Migrants, especially those in precarious legal, residential and economic situations, usually fall outside this imagined user group. While these organizations are aware that surveillance affects migrant and racialized people differently, this rarely translates into sustained engagement with migrant communities. Their contestation takes the form of building privacy infrastructure, lobbying for responsible tech policy, and educating the general public, which is relevant and necessary *per se*, but disconnected from the lived digital vulnerability of people on the move. In this case, specific actions and campaigns such as awareness-raising and digital self-defense workshops, serve as preconditions for resistance but cannot be equated with resistance in a strict sense.

Migrant solidarity organizations, on the other hand, are often overwhelmed by urgent needs that require their immediate action. Faced with increasingly restrictive migration policies, growing xenophobia, and shrinking civic space, they primarily engage in assisting migrants with navigating restrictive border regimes and prioritizing urgent and immediate needs. Issues such as data extraction, digital surveillance, or algorithmic decision-making are often recognized as important but remain secondary in practice. Barriers to engagement are related to the lack of technical knowledge and the absence of time and capacity to address issues that do not pose an immediate threat to life and legal status. Still, these organizations demonstrate interest in the topic, particularly when framed through the lens of racial profiling, labor discrimination, or reorganization of bureaucracy, which includes new technological solutions.

The third organizational type, which is represented by organizations with a long history of building a critical perspective on economic inequalities internationally, offers a promising space for coalition-building and action on different fronts. Their work represents a form of contestation, as they attempt to politicize digitalization and insert civil society into decision-making arenas. Through coalition-building and technical counter-expertise, they move beyond awareness-raising and navigation to engage in structural resistance to neoliberal logics of digitalization. Although the possibility of contestation at different stages and levels is crucial (Alfrink et al., 2023; Lyons et al., 2021) to guarantee democratic rights, the capacity to foster and sustain coalition-building is crucial to participate in such a possibility of algorithmic contestation.

More specifically, the third organizational type specified in our empirical analysis highlights that contestability requires techno-social conditions of possibility: access to information, funding, and legitimacy for citizen participation, shared vocabularies across sectors, and the creation of new civic publics who understand digitalization as a political terrain. These aspects seem pivotal to build collective critical capacity as contestability is not only a matter of “inserting a human into the loop of harmful, biased, or discriminatory technology” (Sarra, 2020).

Regarding public opinion, the speed and scale of digital transformation in migration governance is not easy to mobilize against. While data extraction and automated decision-making tools are becoming increasingly embedded in asylum procedures, border control, and migration governance in general, the capacity to scrutinize, resist, or even form a critical opinion on these processes remains extremely difficult. High levels of institutional trust, entrenched techno-optimism, and the normalization of digital state infrastructure, particularly in Scandinavian countries where the digitalization of public services is an important priority, appear as some of the important factors.

Together, the different repertories of the three organizational types open space to analyze contestation not as a singular or heroic act, but as a field of constrained and creative responses. They shed light on how resistance to automated systems can emerge in uneven, sometimes fragmented ways, through legal challenges, alternative forms of care, information sharing, or temporary alliances across civil society. Rather than idealizing resistance or assuming equal capacity to act, our analysis situates the (im)possibility of algorithmic contestation within the complex and unequal terrain of migration governance, considering how the distribution of agency is also shaped by power, access, and positionality. This condition applies also to actors participating in civil society organizations.

## Conclusions

This article has examined how Scandinavian civil society organizations engage with the challenges of digitalization in the field of migration governance. Across different organizational profiles—from established migrant solidarity networks to hybrid organizations explicitly addressing democratic digitalization—a recurrent tension emerges between analog urgencies and the growing awareness of digital risks. While resource constraints, activist fatigue, and dependency on international networks are common across Europe, the Scandinavian context highlights particular dynamics.

First, the high levels of institutional trust and embeddedness of civil society in state structures shape how digital issues are addressed. Rather than contesting state authority directly, organizations in Norway and Sweden often act through dialogue, coalition-building, and knowledge-sharing. This proximity, however, can limit their autonomy, leaving them ill-equipped to respond rapidly to new forms of algorithmic governance. Second, the Scandinavian digital rights field is strikingly underdeveloped, with only a handful of organizations explicitly combining technological expertise with social justice concerns. This absence contrasts with other European contexts and contributes to the marginalization of migrants' digital vulnerabilities. Third, migration politics have undergone a profound shift since 2015, as restrictive asylum regimes and normalized xenophobic discourse reduce mobilization capacity, divert attention away from digital risks, and foster a sense of disempowerment among activists.

Taken together, these features suggest that Scandinavian civil society occupies an ambivalent position: equipped with strong traditions of coalition-building and public legitimacy, but simultaneously constrained by high institutional trust, dependency on state funding, and the shrinking political space for solidarity with migrants. This ambivalence has important implications for democratic governance. If civil society actors are unable to meaningfully contest the digital infrastructures that mediate migration control, the region risks sliding into what some scholars call “creeping autocratization”—a gradual erosion of rights and accountability not through spectacular authoritarian measures, but through opaque technologies embedded in everyday bureaucratic practice.

By situating Scandinavian cases within the broader European landscape, our findings underscore that the future of contestability in migration governance will depend not only on technical expertise but also on rethinking the role of civil society in highly digitalized democracies. Norway and Sweden, with their strong democratic traditions yet rapidly hardening migration regimes, are critical sites for observing how digital technologies may reconfigure the balance between state power, migrant rights, and democratic participation.

## Data Availability

The datasets presented in this article are not readily available because of confidentiality reasons. Requests to access the datasets should be directed to the corresponding author.

## Appendix

**Table A1:** Empirical data sources.

**Code**	**Country**	**Type of organization**	**Material**
O1	Sweden	Digital rights	Interview with a volunteer and member board. Website
O2	Norway	Digital rights	Website, Facebook and Twitter
O3	Norway	Migrant rights	Interview with a staff member. Website
O4	Norway	Human rights, focusing on environment, digitalization	Interview with the CEO. Interview with a working group leader. Website and press articles and interviews
O5	Sweden	Digital rights	Website
O6	Sweden	Digital rights	Website, Facebook account and online events
O7	Sweden	Migrant rights	Website and Facebook account
08	Sweden	Migrant rights	Facebook and Instagram accounts
